# (*E*)-4-Bromo-*N*′-(2-hydr­oxy-1-naphthyl­methyl­ene)benzohydrazide

**DOI:** 10.1107/S1600536808031395

**Published:** 2008-10-04

**Authors:** Yun-Peng Diao, Qi-Hui Zhang, Da-Cheng Wang, Xu-Ming Deng

**Affiliations:** a College of Animal Science and Veterinary Medicine, Jilin University, Jilin 130062, People’s Republic of China; b College of Pharmacy, Dalian Medical University, Dalian 116044, People’s Republic of China; c School of Traditional Chinese Materia Medica, Shenyang Pharmaceutical University, Shenyang 110016, People’s Republic of China

## Abstract

The title compound, C_18_H_13_BrN_2_O_2_, was synthesized by the reaction of 2-hydr­oxy-1-naphthaldehyde with 4-bromo­benzohydrazide. This Schiff base mol­ecule has an *E* configuration about the C=N bond and is almost planar, the dihedral angle between the mean planes through the substituted benzene ring and the naphthyl system being 6.6 (2)°. There is an intra­molecular O—H⋯N hydrogen bond involving the naphthyl hydr­oxy substituent and the N′ atom of the hydrazide group. In the crystal structure, mol­ecules are linked through inter­molecular N—-H⋯O hydrogen bonds to form chains extending along the *b* direction.

## Related literature

For related structures, see: Brückner *et al.* (2000 ▶); Diao (2007 ▶); Diao *et al.* (2007 ▶, 2008 ▶); Harrop *et al.* (2003 ▶); Huang *et al.* (2007 ▶); Li *et al.* (2007 ▶); Ren *et al.* (2002 ▶).
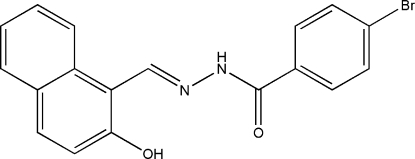


## Experimental

### 

#### Crystal data

C_18_H_13_BrN_2_O_2_
*M* *_r_* = 369.21
Monoclinic, 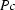

*a* = 6.185 (2) Å
*b* = 4.7638 (19) Å
*c* = 25.689 (10) Å
β = 95.449 (7)°
*V* = 753.5 (5) Å^3^
*Z* = 2
Mo *K*α radiation
μ = 2.74 mm^−1^
*T* = 298 (2) K
0.30 × 0.30 × 0.28 mm

#### Data collection

Bruker SMART CCD area-detector diffractometer
Absorption correction: multi-scan (*SADABS*; Bruker, 2000 ▶) *T*_min_ = 0.494, *T*_max_ = 0.514 (expected range = 0.446–0.464)
5817 measured reflections
3119 independent reflections
2443 reflections with *I* > 2σ(*I*)
*R*_int_ = 0.034

#### Refinement

*R*[*F* ^2^ > 2σ(*F* ^2^)] = 0.040
*wR*(*F* ^2^) = 0.101
*S* = 0.90
3119 reflections
212 parameters
3 restraints
H atoms treated by a mixture of independent and constrained refinement
Δρ_max_ = 0.31 e Å^−3^
Δρ_min_ = −0.24 e Å^−3^
Absolute structure: Flack (1983 ▶), 1493 Friedel pairs
Flack parameter: 0.026 (12)

### 

Data collection: *SMART* (Bruker, 2000 ▶); cell refinement: *SAINT* (Bruker, 2000 ▶); data reduction: *SAINT*; program(s) used to solve structure: *SHELXTL* (Sheldrick, 2008 ▶); program(s) used to refine structure: *SHELXTL*; molecular graphics: *SHELXTL*; software used to prepare material for publication: *SHELXTL*.

## Supplementary Material

Crystal structure: contains datablocks global, I. DOI: 10.1107/S1600536808031395/su2066sup1.cif

Structure factors: contains datablocks I. DOI: 10.1107/S1600536808031395/su2066Isup2.hkl

Additional supplementary materials:  crystallographic information; 3D view; checkCIF report

## Figures and Tables

**Table 1:** Hydrogen-bond geometry (Å, °)

*D*—H⋯*A*	*D*—H	H⋯*A*	*D*⋯*A*	*D*—H⋯*A*
N1—H1⋯O1^i^	0.89 (3)	1.99 (3)	2.841 (4)	160 (6)
O2—H2⋯N2	0.82	1.86	2.580 (4)	145

Symmetry code: (i) 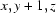.

**Fig. 1.**
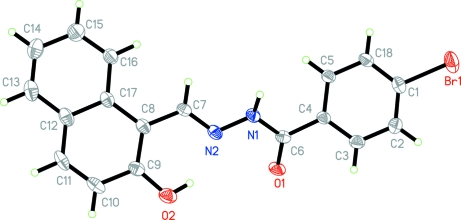
The molecular structure of the title compound with displacement parameters drawn at the 30% probability level.

**Fig. 2.**
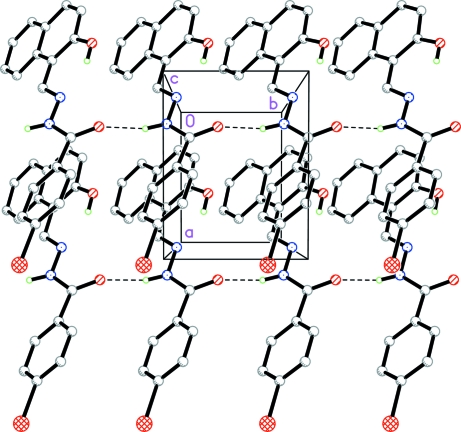
A persepctive view along the a axis of the crystal packing of the title compound.

## supplementary crystallographic information

### Comment

Schiff base compounds have been found to have potential pharmacological and antitumor properties (Brückner *et al.*, 2000; Harrop *et al.*, 2003; Ren *et al.*, 2002). Recently, a few Schiff base compounds obtained from the reaction of aldehydes with benzohydrazides have been reported (Diao *et al.*, 2008; Diao *et al.*, 2007; Diao, 2007; Li *et al.*, 2007; Huang *et al.*, 2007). As a further study of such compounds, we report here on the structure of the title compound.

The title compound, a Schiff base synthesized by the reaction of 2-hydroxy-1-naphthaldehyde with 4-bromobenzohydrazide, is almost planar (Fig. 1), with the dihedral angle between the mean planes of the substituted benzene ring and the naphthyl ring being only 6.6 (2)°. The torsion angles C4—C6—N1—N2 and C8—C7—N2—N1 are 0.9 (3) and 2.9 (3)°, respectively. There is an intramolecular O-H···N hydrogen bond involving the naphthyl hydroxyl substituent and the NH H-atom of the hydrazide group (Table 1).

In the crystal molecules are linked via N–H···O intermolecular hydrogen bonds (Table 1), to form chains extending in the *b* direction (Fig. 2).

### Experimental

4-Bromobenzaldehyde (0.1 mmol, 18.5 mg) and 2-hydroxy-1-naphthaldehyde (0.1 mmol, 17.2 mg) were dissolved in methanol (20 ml). The mixture was stirred at reflux for 1 h and cooled to room temperature. After keeping the solution in air for alomost two weeks, yellow block-like crystals of the title compound were formed.

### Refinement

Atom H1 was located from a difference Fourier map and refined isotropically, with the N–H distance restrained to 0.90 (1) Å. The other H atoms were placed in calculated positions and treated as riding atoms, with C–H = 0.93 Å, O–H = 0.82 Å, and *U*_iso_(H) = 1.2*U*_eq_(C) and 1.5*U*_eq_(O).

### Crystal data

**Table:**

C_18_H_13_BrN_2_O_2_	*F*(000) = 372
*M**_r_* = 369.21	*D*_x_ = 1.627 Mg m^−^^3^
Monoclinic, *P**c*	Mo *K*α radiation, λ = 0.71073 Å
*a* = 6.185 (2) Å	Cell parameters from 1589 reflections
*b* = 4.7638 (19) Å	θ = 2.6–24.5°
*c* = 25.689 (10) Å	µ = 2.74 mm^−^^1^
β = 95.449 (7)°	*T* = 298 K
*V* = 753.5 (5) Å^3^	Block, yellow
*Z* = 2	0.30 × 0.30 × 0.28 mm

### Data collection

**Table:**

Bruker SMART CCD area-detector diffractometer	3119 independent reflections
Radiation source: fine-focus sealed tube	2443 reflections with *I* > 2σ(*I*)
graphite	*R*_int_ = 0.034
ω scans	θ_max_ = 27.0°, θ_min_ = 1.6°
Absorption correction: multi-scan (SADABS; Bruker, 2000)	*h* = −7→7
*T*_min_ = 0.494, *T*_max_ = 0.514	*k* = −6→6
5817 measured reflections	*l* = −32→32

### Refinement

**Table:**

Refinement on *F*^2^	Secondary atom site location: difference Fourier map
Least-squares matrix: full	Hydrogen site location: inferred from neighbouring sites
*R*[*F*^2^ > 2σ(*F*^2^)] = 0.040	H atoms treated by a mixture of independent and constrained refinement
*wR*(*F*^2^) = 0.101	*w* = 1/[σ^2^(*F*_o_^2^) + ]
*S* = 0.90	(Δ/σ)_max_ = 0.001
3119 reflections	Δρ_max_ = 0.31 e Å^−^^3^
212 parameters	Δρ_min_ = −0.24 e Å^−^^3^
3 restraints	Absolute structure: Flack (1983), 1493 Friedel pairs
Primary atom site location: structure-invariant direct methods	Flack parameter: 0.026 (12)

### Special details

**Table:**

Geometry. All e.s.d.'s (except the e.s.d. in the dihedral angle between two l.s. planes) are estimated using the full covariance matrix. The cell e.s.d.'s are taken into account individually in the estimation of e.s.d.'s in distances, angles and torsion angles; correlations between e.s.d.'s in cell parameters are only used when they are defined by crystal symmetry. An approximate (isotropic) treatment of cell e.s.d.'s is used for estimating e.s.d.'s involving l.s. planes.
Refinement. Refinement of *F*^2^ against ALL reflections. The weighted *R*-factor *wR* and goodness of fit *S* are based on *F*^2^, conventional *R*-factors *R* are based on *F*, with *F* set to zero for negative *F*^2^. The threshold expression of *F*^2^ > σ(*F*^2^) is used only for calculating *R*-factors(gt) *etc*. and is not relevant to the choice of reflections for refinement. *R*-factors based on *F*^2^ are statistically about twice as large as those based on *F*, and *R*- factors based on ALL data will be even larger.

### Fractional atomic coordinates and isotropic or equivalent isotropic displacement parameters (Å^2^)

**Table:**

	*x*	*y*	*z*	*U*_iso_*/*U*_eq_	
Br1	1.08310 (11)	0.22527 (11)	1.13545 (4)	0.0685 (2)	
O1	0.2012 (5)	−0.3701 (5)	0.99811 (12)	0.0474 (7)	
O2	−0.3678 (6)	−0.2844 (6)	0.91908 (13)	0.0509 (8)	
H2	−0.2458	−0.2481	0.9327	0.076*	
N1	0.1759 (5)	0.0647 (6)	0.96274 (13)	0.0379 (7)	
N2	−0.0057 (5)	−0.0129 (6)	0.93167 (12)	0.0377 (7)	
C18	0.7851 (7)	0.2520 (7)	1.04481 (17)	0.0407 (9)	
H18	0.8775	0.3902	1.0342	0.049*	
C1	0.8295 (7)	0.1191 (9)	1.09188 (16)	0.0433 (9)	
C2	0.6996 (7)	−0.0854 (9)	1.10869 (16)	0.0480 (10)	
H2A	0.7329	−0.1711	1.1410	0.058*	
C3	0.5173 (7)	−0.1632 (8)	1.07685 (17)	0.0432 (10)	
H3	0.4282	−0.3052	1.0875	0.052*	
C4	0.4659 (6)	−0.0328 (7)	1.02954 (15)	0.0340 (8)	
C5	0.6012 (6)	0.1770 (7)	1.01362 (16)	0.0369 (8)	
H5	0.5673	0.2669	0.9818	0.044*	
C6	0.2705 (6)	−0.1305 (8)	0.99609 (15)	0.0350 (8)	
C7	−0.0776 (6)	0.1545 (8)	0.89501 (16)	0.0357 (8)	
H7	−0.0023	0.3190	0.8893	0.043*	
C8	−0.2727 (6)	0.0903 (7)	0.86278 (15)	0.0361 (8)	
C9	−0.4138 (6)	−0.1175 (8)	0.87700 (16)	0.0399 (9)	
C10	−0.6139 (7)	−0.1627 (9)	0.8477 (2)	0.0513 (11)	
H10	−0.7084	−0.2975	0.8587	0.062*	
C11	−0.6716 (6)	−0.0146 (9)	0.80394 (19)	0.0527 (11)	
H11	−0.8065	−0.0462	0.7857	0.063*	
C12	−0.5319 (7)	0.1866 (8)	0.78536 (19)	0.0459 (10)	
C13	−0.5851 (8)	0.3322 (10)	0.7382 (2)	0.0558 (12)	
H13	−0.7177	0.2963	0.7191	0.067*	
C14	−0.4491 (8)	0.5229 (10)	0.71980 (18)	0.0596 (12)	
H14	−0.4861	0.6156	0.6883	0.071*	
C15	−0.2513 (8)	0.5774 (10)	0.74925 (17)	0.0568 (11)	
H15	−0.1576	0.7101	0.7372	0.068*	
C16	−0.1927 (6)	0.4422 (8)	0.79472 (15)	0.0441 (9)	
H16	−0.0587	0.4806	0.8129	0.053*	
C17	−0.3314 (6)	0.2444 (7)	0.81494 (17)	0.0377 (9)	
H1	0.209 (11)	0.245 (4)	0.969 (3)	0.080*	

### Atomic displacement parameters (Å^2^)

**Table:**

	*U*^11^	*U*^22^	*U*^33^	*U*^12^	*U*^13^	*U*^23^
Br1	0.0451 (2)	0.0994 (4)	0.0583 (3)	−0.0070 (3)	−0.00903 (17)	−0.0151 (3)
O1	0.0489 (17)	0.0246 (13)	0.068 (2)	−0.0093 (12)	−0.0010 (14)	0.0020 (13)
O2	0.055 (2)	0.0432 (16)	0.055 (2)	−0.0164 (13)	0.0070 (15)	−0.0067 (14)
N1	0.0431 (18)	0.0268 (15)	0.0425 (18)	−0.0060 (14)	−0.0022 (14)	−0.0048 (13)
N2	0.0343 (15)	0.0308 (15)	0.0473 (19)	−0.0038 (13)	0.0002 (13)	−0.0074 (14)
C18	0.036 (2)	0.043 (2)	0.044 (2)	−0.0092 (17)	0.0070 (17)	−0.0058 (17)
C1	0.036 (2)	0.047 (2)	0.046 (3)	−0.0004 (18)	0.0002 (17)	−0.0128 (19)
C2	0.050 (2)	0.050 (3)	0.042 (2)	0.000 (2)	−0.0038 (19)	0.0053 (19)
C3	0.044 (2)	0.035 (2)	0.051 (3)	−0.0049 (17)	0.0040 (19)	0.0034 (17)
C4	0.038 (2)	0.0274 (18)	0.037 (2)	−0.0041 (15)	0.0040 (15)	−0.0030 (15)
C5	0.038 (2)	0.0343 (19)	0.038 (2)	−0.0062 (15)	0.0045 (16)	−0.0027 (15)
C6	0.038 (2)	0.0250 (18)	0.043 (2)	−0.0006 (16)	0.0105 (17)	−0.0058 (16)
C7	0.035 (2)	0.0306 (18)	0.041 (2)	−0.0062 (15)	0.0040 (16)	−0.0041 (16)
C8	0.036 (2)	0.0308 (18)	0.041 (2)	−0.0011 (16)	0.0023 (16)	−0.0111 (16)
C9	0.041 (2)	0.035 (2)	0.044 (2)	−0.0055 (17)	0.0084 (17)	−0.0101 (17)
C10	0.038 (2)	0.049 (2)	0.068 (3)	−0.0138 (19)	0.008 (2)	−0.015 (2)
C11	0.031 (2)	0.060 (3)	0.064 (3)	−0.0061 (18)	−0.0082 (18)	−0.023 (2)
C12	0.038 (2)	0.044 (2)	0.055 (3)	0.0017 (17)	−0.0037 (19)	−0.0178 (18)
C13	0.049 (3)	0.057 (3)	0.057 (3)	0.009 (2)	−0.013 (2)	−0.015 (2)
C14	0.067 (3)	0.068 (3)	0.043 (3)	0.010 (3)	−0.003 (2)	−0.001 (2)
C15	0.059 (3)	0.066 (3)	0.046 (3)	−0.002 (2)	0.008 (2)	−0.001 (2)
C16	0.038 (2)	0.051 (3)	0.044 (2)	−0.0058 (18)	0.0023 (17)	−0.0005 (19)
C17	0.033 (2)	0.036 (2)	0.043 (2)	0.0018 (15)	0.0005 (16)	−0.0104 (16)

### Geometric parameters (Å, °)

**Table:**

Br1—C1	1.907 (4)	C7—C8	1.430 (5)
O1—C6	1.222 (5)	C7—H7	0.9300
O2—C9	1.350 (5)	C8—C9	1.391 (5)
O2—H2	0.8200	C8—C17	1.448 (6)
N1—C6	1.359 (5)	C9—C10	1.403 (6)
N1—N2	1.366 (4)	C10—C11	1.346 (7)
N1—H1	0.89 (3)	C10—H10	0.9300
N2—C7	1.281 (5)	C11—C12	1.404 (6)
C18—C1	1.369 (6)	C11—H11	0.9300
C18—C5	1.375 (6)	C12—C13	1.407 (7)
C18—H18	0.9300	C12—C17	1.419 (6)
C1—C2	1.359 (6)	C13—C14	1.354 (7)
C2—C3	1.379 (6)	C13—H13	0.9300
C2—H2A	0.9300	C14—C15	1.400 (7)
C3—C4	1.375 (5)	C14—H14	0.9300
C3—H3	0.9300	C15—C16	1.353 (6)
C4—C5	1.389 (5)	C15—H15	0.9300
C4—C6	1.490 (5)	C16—C17	1.406 (6)
C5—H5	0.9300	C16—H16	0.9300
			
C9—O2—H2	109.5	C9—C8—C17	118.1 (3)
C6—N1—N2	117.7 (3)	C7—C8—C17	120.8 (3)
C6—N1—H1	118 (5)	O2—C9—C8	122.7 (4)
N2—N1—H1	121 (5)	O2—C9—C10	116.6 (4)
C7—N2—N1	118.0 (3)	C8—C9—C10	120.8 (4)
C1—C18—C5	118.7 (4)	C11—C10—C9	121.2 (4)
C1—C18—H18	120.7	C11—C10—H10	119.4
C5—C18—H18	120.7	C9—C10—H10	119.4
C2—C1—C18	122.5 (4)	C10—C11—C12	121.3 (4)
C2—C1—Br1	118.8 (3)	C10—C11—H11	119.4
C18—C1—Br1	118.7 (3)	C12—C11—H11	119.4
C1—C2—C3	118.6 (4)	C11—C12—C13	121.9 (4)
C1—C2—H2A	120.7	C11—C12—C17	119.0 (4)
C3—C2—H2A	120.7	C13—C12—C17	119.1 (4)
C4—C3—C2	120.7 (4)	C14—C13—C12	121.9 (4)
C4—C3—H3	119.7	C14—C13—H13	119.0
C2—C3—H3	119.7	C12—C13—H13	119.0
C3—C4—C5	119.3 (4)	C13—C14—C15	118.4 (4)
C3—C4—C6	118.4 (3)	C13—C14—H14	120.8
C5—C4—C6	122.2 (4)	C15—C14—H14	120.8
C18—C5—C4	120.2 (4)	C16—C15—C14	121.8 (5)
C18—C5—H5	119.9	C16—C15—H15	119.1
C4—C5—H5	119.9	C14—C15—H15	119.1
O1—C6—N1	122.3 (3)	C15—C16—C17	121.0 (4)
O1—C6—C4	122.4 (3)	C15—C16—H16	119.5
N1—C6—C4	115.3 (3)	C17—C16—H16	119.5
N2—C7—C8	120.5 (3)	C16—C17—C12	117.7 (4)
N2—C7—H7	119.8	C16—C17—C8	122.7 (4)
C8—C7—H7	119.8	C12—C17—C8	119.5 (4)
C9—C8—C7	121.1 (4)		

### Hydrogen-bond geometry (Å, °)

**Table:**

*D*—H···*A*	*D*—H	H···*A*	*D*···*A*	*D*—H···*A*
N1—H1···O1^i^	0.89 (3)	1.99 (3)	2.841 (4)	160 (6)
O2—H2···N2	0.82	1.86	2.580 (4)	145

Symmetry codes: (i) *x*, *y*+1, *z*.
